# Global health: time to move beyond the USA?

**DOI:** 10.1016/j.lansea.2025.100577

**Published:** 2025-04-10

**Authors:** The Lancet Regional Health – Southeast Asia

2025 marks 77 years since the founding of WHO on April 7, 1948. This founding conferred WHO with the responsibility of global health governance. The USA was one of the co-founders and major funders of WHO. Together, WHO and the USA pioneered the work of coordinating scientific evidence to advance global health, fostering cooperation among countries, agencies, and regions, based on the core values of public health—equity, justice, and human rights. The COVID-19 pandemic was one of the defining moments in the history of WHO, that questioned its relevance in global health governance. Today, WHO once again faces a major crisis with withdrawal of one of its major funders. While the world grapples with measures to mitigate the impacts of the USA's disengagement from international development and multilateral organisations, we use this opportunity to reflect beyond chaos and crisis, and see how we can set a new trend of self-reliance and national and regional collaboration in southeast Asia.

South Asian countries, heavily dependent on international aid for diseases like malaria, HIV, and tuberculosis, could face setbacks in control efforts. India might be less affected due to minimal reliance on US funding, but countries like Myanmar, Bangladesh, and Afghanistan may suffer due to programme disruptions and funding shortages, risking lives and health security. In Afghanistan, 167 health facilities had shut down due to funding shortages, cutting off lifesaving medical care to 1.6 million people across 25 provinces. Global health advocates have been suggesting quick fixes to help fill funding gaps created by the US withdrawal, such as collaboration with the private sector, increasing role of NGOs in providing health care services, seeking support from philanthropic organisations, and other countries stepping in for the USA (like Canada, which recently announced an allocation of US $272.1 million for Bangladesh and the Indo-Pacific region). Many have called upon other alliances such as BRICS and G20 countries to take up the space left by the USA.

This current hullabaloo also opens up discussions about the ethics of withdrawal of a country funding humanitarian aid and health projects in some of the poorest and most conflict-ridden countries of the world. The time has come for multilateral agencies to come together to consider creating a convention on ethics for external aid on health, similar to what has been achieved through the Declaration of Helsinki on medical research involving human participants. The Global Fund to Fight AIDS, Tuberculosis and Malaria—the largest donor to health programmes for these three diseases—has set the exemplary ethical standard of continuation of services for patients under treatment or preventive services even when the grant is withdrawn for performance failures in the recipient country. Recent events demand that these standards are made compulsory and institutionalised in each country system in the event of withdrawal of foreign aid. After all, delivering humanitarian aid and health to all will require transparency and accountability at all levels, including countries that provide funding and ones that receive it.

The second opportunity, and a tremendous one, is for countries from the southeast Asia region to leverage their manufacturing and technical capacities and innovation ecosystems to support WHO in addressing global health challenges. Countries like Thailand, Sri Lanka, India, Bangladesh, and Indonesia have best practices to offer in capacity building and empowering other countries dependent on foreign aid for their meeting own health system needs. Together, these countries—particularly the larger countries like India, Bangladesh, and Indonesia—could use their substantial capacities to assume a larger role in global health and establish organisations to help and support countries in various regions of the world modelled after international development agencies.

Finally, this moment also calls for restoring WHO's value and strengthening its independence. Despite being a democratic UN body, WHO remains vulnerable to political and financial pressures. The current political ethos does not allow WHO to achieve its full potential—to empower nations to and build equitable health systems that suit their health needs, in all countries of the world—thereby threatening the core values of its founding. To elevate WHO to its rightful status, countries must unite to empower WHO, independent of political and financial influences, with statutory authority to direct governments in taking necessary actions to protect the health and wellbeing of all, while also respecting country sovereignty. We must not forget that we will need a stronger WHO to lead countries through the next pandemic, which may be right around the corner.

